# CD47 deficiency in tumor stroma promotes tumor progression by enhancing angiogenesis

**DOI:** 10.18632/oncotarget.9899

**Published:** 2016-06-07

**Authors:** Lu Gao, Kexin Chen, Qi Gao, Xiaodan Wang, Jian Sun, Yong-Guang Yang

**Affiliations:** ^1^ The First Hospital and Institute of Immunology, Jilin University, Changchun, China; ^2^ Columbia Center for Translational Immunology, Columbia University College of Physicians and Surgeons, New York, New York, USA

**Keywords:** angiogenesis, CD47, SIRPα, TSP1, tumorigenesis

## Abstract

CD47 is a transmembrane protein that functions as a receptor for thrombospondin-1 (TSP1) and a ligand for inhibitory receptor signal-regulatory protein-α (SIRPα). Blocking the interaction between CD47 on tumor cells and SIRPα on macrophages has been shown to induce antitumor responses. Here we investigated the role of CD47 expression in tumor stroma in tumorigenesis by comparing tumor growth in wild-type (WT) and CD47-deficient mice after subcutaneous injection of syngeneic prostate cancer cells. We found that CD47 deficiency in tumor stromal endothelial cells enhances angiogenesis, leading to suppressed tumor necrosis formation and accelerated tumor progression. Tumors from CD47-deficient mice also showed improved vascular integrity and stability, as well as increased expression of vascular endothelial growth factor (VEGF)-A and VEGF receptor 2 (VEGFR2) compared to those from WT mice. Moreover, reduced macrophage recruitment, likely due to decreased TSP1 production, was detected in tumors from CD47-deficient mice. Our results indicate that although treatment with antibody against CD47 induces antitumor immune responses by blocking the inhibitory CD47-SIRPα signaling, this treatment may also potentially promote tumor progression by blocking CD47 signaling in tumor stromal endothelial cells.

## INTRODUCTION

Angiogenesis is a key process in the progression of cancer from an *in situ* lesion to an invasive and metastatic disease, providing the basis for the development of anti-angiogenic therapies [1, 2]. Vascular endothelial growth factor A (VEGF-A) is a major player in angiogenesis among the VEGF family that promotes angiogenesis through binding and activating the tyrosine kinase receptors, VEGFR1 (Flt-1) and VEGFR2 (KDR/Flk-1) [3, 4]. CD47 (also known as integrin-associated protein; IAP) is a transmembrane protein of the immunoglobulin superfamily that serves as a receptor for thrombospondin-1 (TSP1) [5]. Interaction of TSP1 with CD47 inhibits NO-signaling in endothelial cells as well as vascular smooth muscle cells and platelets [6, 7], mainly by inhibiting VEGFR2 [8]. TSP1 was also reported to suppress angiogenesis. It has been shown that TSP1 inhibits angiogenic responses by directly binding to VEGF [9]. Moreover, TSP1 was found to inhibit VEGFR2 signaling by disrupting the association of CD47 with VEGFR2 [10], suggesting that CD47 is involved in TSP1-mediated inhibition of angiogenesis.

CD47 also serves as a ligand of the inhibitory receptor, signal-regulatory protein-α (SIRPα), expressed on macrophages and dendritic cells (DCs). Binding of CD47 with SIRPα on macrophages and DCs conveys a “don't eat me” signal [11–14]. CD47 expression is elevated in varying cancer cells, and injection of blocking antibodies against CD47 to block CD47-SIRPα signaling has been shown to induce antitumor immune responses [15–18]. However, it remains unclear as to whether and how administration of anti-CD47 antibody may affect tumor progression via its potential role in the regulation of angiogenesis.

Here we investigated the role of CD47 expression in non-tumor stromal cells in tumorigenesis by comparing tumor angiogenesis and progression in wild-type (WT) and CD47-deficient mice after injection of syngeneic cancer cells. We show that the lack of CD47 in tumor stromal cells promotes angiogenesis and enhances vascular integrity and stability, leading to accelerated tumor progression. Furthermore, increased expression of VEGF-A and VEGFR2 was detected in tumors from CD47-deficient mice compared to those from WT mice. These results suggest that targeting CD47 by anti-CD47 antibody might promote angiogenesis by blocking TSP1-CD47 signaling and hence, enhancing tumor cell survival.

## RESULTS

### Enhanced tumor growth without apparent necrosis in CD47-deficient mice

**Figure 1:**
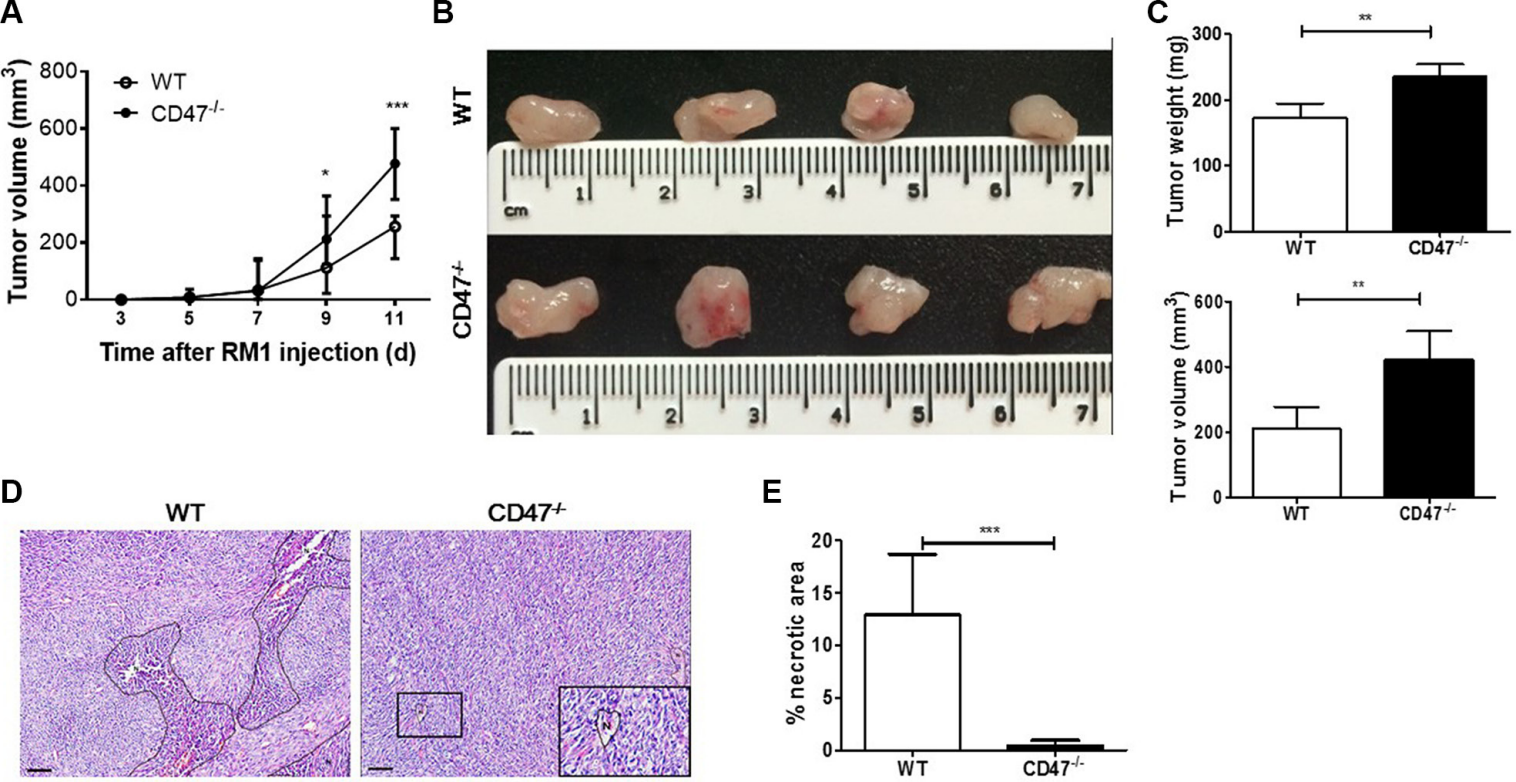
Enhanced tumor growth without apparent necrosis in mice lacking CD47 (**A**) Growth rates of tumors from WT and CD47^−/−^ mice (*n* = 6 per group). (**B**–**E**) Characterization of tumors removed from WT and CD47^−/−^ mice at day 11 (*n* = 4 per group). (B) Gross morphology. (C) Tumor weight (upper) and volume (lower). (D) H&E staining of tumor samples (Scale bar, 200 μm). N, necrosis. (E) Percentages of necrotic area in tumors. Eight randomly selected fields (100×) were analyzed and the percentage of necrotic areas was determined using Image-Pro Plus 6.0 software. Data are mean ± SDs. *P < 0.05, **P < 0.01; ***P < 0.001.

Angiogenesis is critical to tumor progression and torrid growth results in necrosis. Thus, to test our hypothesis that CD47 deficiency in tumor endothelial cells may enhance angiogenesis, we first assessed the growth and central necrosis in the tumors developed in WT vs. CD47-deficient mice following subcutaneous injection of syngeneic RM1 prostate cancer cells. Although the two groups of mice had similar incidence and latency period for palpable tumor formation, RM1 tumor grew significantly more aggressively in CD47-deficient than in WT mice (Figure 1A). In a separate experiment, we surgically removed the tumors 11 days after inoculation and found vascularized tumors in both WT and CD47-deficient mice (Figure 1B). However, the weight and volume of the tumors from CD47-deficient mice were significantly greater than those of the WT mice (Figure 1C). Necrotic lesions were readily detected in the tumors from WT mice, but were almost undetectable in those from CD47-deficient mice (Figure 1D and 1E). These data indicate that tumor cells exhibited an enhanced potential to grow and survive in mice lacking CD47 than in WT mice.

### Tumors from CD47-deficient mice exhibited improved angiogenesis and vascular integrity than those from WT mice

**Figure 2:**
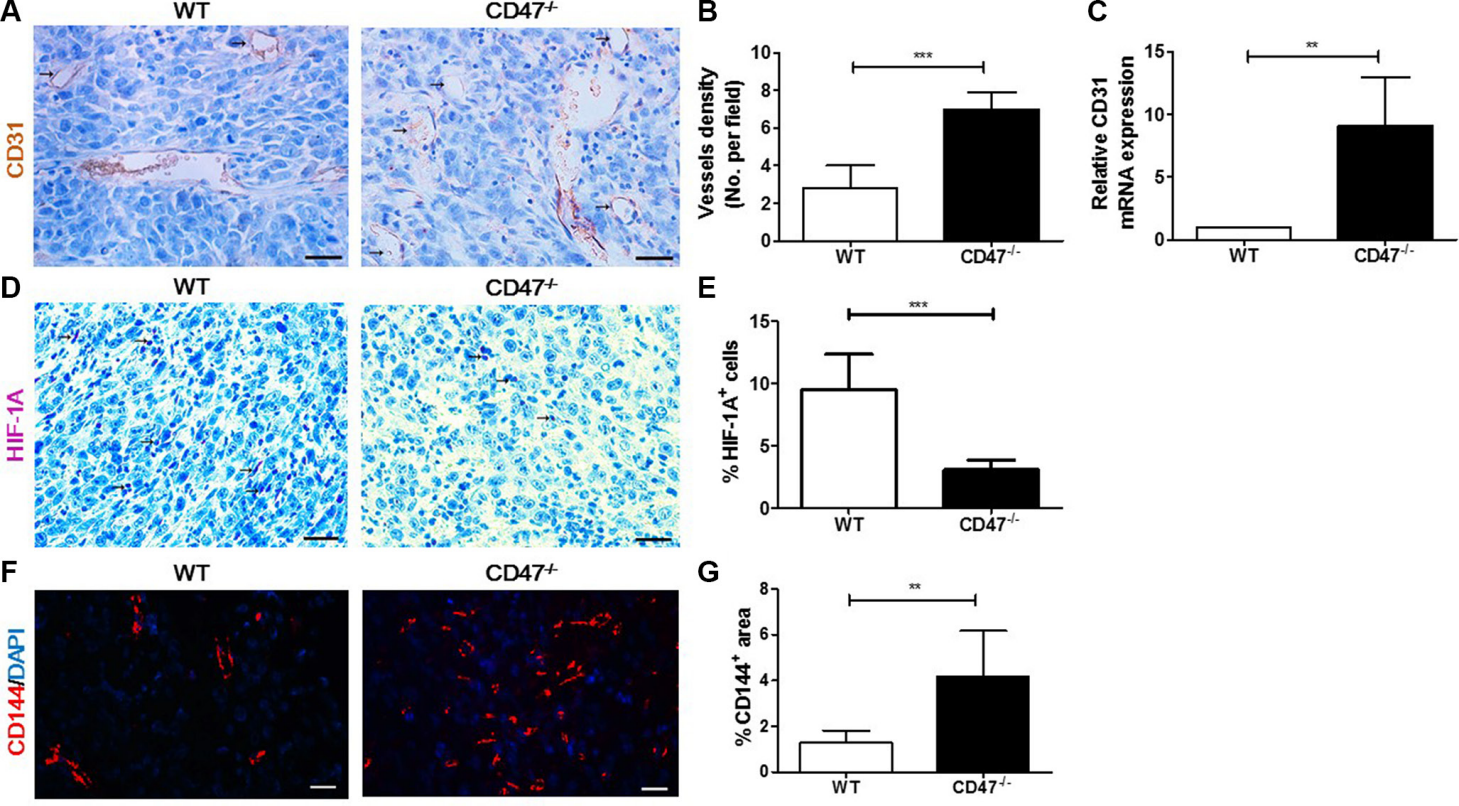
Tumors from CD47-deficient mice exhibited improved tumor angiogenesis and vascular integrity compared to those from WT mice Tumors were surgically removed from WT or CD47^−/−^ mice 11 days after tumor cell injection for the following analyses (*n* = 4 per group). (**A**) Representative images of CD31 staining (black arrow) of tumor sections (Scale bar, 20 μm). (**B**) The microvessel density (MVD) quantified by counting positive cells in six randomly selected fields (400×) using Image Pro Plus 6.0 software. (**C**) CD31 mRNA levels in tumors quantified by real-time qPCR. (**D**) Representative images of HIF-1A staining (pink) of tumor sections (Scale bar, 20 μm). (**E**) Percentages of HIF-1A^+^ cells in tumors quantified by counting positive cells in six randomly selected fields (400×) using Image Pro Plus 6.0 software. (**F**) Representative images of CD144 staining (red) of tumor sections (Scale bar, 20 μm). (**G**) Percentages of CD144^+^ area in tumors quantified using Image Pro Plus 6.0 software. Data are mean ± SDs. **P < 0.01, ***P < 0.001.

To determine whether enhanced tumor growth and reduced tumor necrosis formation in mice lacking CD47 were due to greater angiogenic potential of CD47-deficient endothelial cells, we next measured the microvessel density (MVD) by CD31 staining in tumor tissues from WT and CD47-deficient mice. MVD is a measurement of cancer angiogenesis, which is used as a predictor of metastasis and survival [19, 20]. Tumors in CD47-deficient mice exhibited an increased number of vessels compared to those in WT mice (Figure 2A and 2B). Consistently, there was significantly increased expression of CD31 mRNA in tumors from CD47-deficient than in those from WT mice (Figure 2C). Malignant tumors often outgrow their blood supply, resulting in hypoxia and formation of central necrosis [21]. Thus, we measured the level of hypoxia-inducible factor (HIF)-1A, a specific marker of hypoxia [22], in tumor tissues by IHC. As shown in Figure 2D and 2E, the levels of HIF-1A in the tumors of CD47-deficient mice were significantly lower than in those from WT mice. These results clearly showed that tumors grown in mice lacking CD47 have enhanced angiogenesis that promotes tumor growth and prevents hypoxia and the associated tumor necrosis formation.

To test whether CD47 also regulates vascular integrity in cancer tissues, we analyzed vascular endothelial (VE)-cadherin (CD144) expression in the tumor vasculature by immunofluorescence staining. VE-cadherin is known to be crucial for vessel assembly and integrity during angiogenesis [23]. We found that tumors grown in CD47-deficient mice had significantly increased VE-cadherin expression compared to those grown in WT mice (Figure 2F and 2G), suggesting that CD47 may inhibit VE-cadherin expression in endothelial cells. The amalgamation of these results indicates that CD47 deficiency in tumor endothelial cells may promote tumor growth by enhancing their angiogenic potential and tumor vascular integrity and stability.

### Increased VEGF and VEGFR2 expression in tumor from CD47-deficient mice

**Figure 3:**
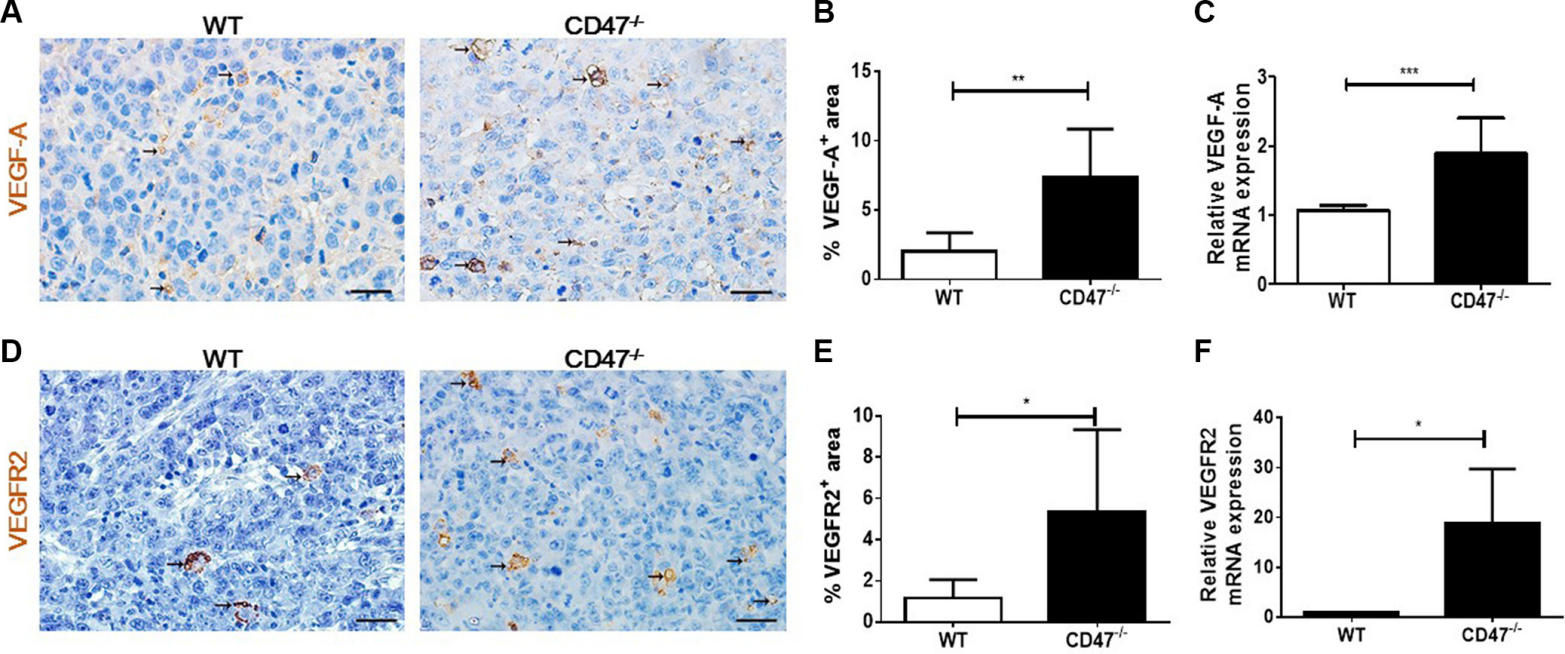
Increased VEGF and VEGFR2 expression in tumors from CD47-deficient mice (**A**) Representative images of tumor sections stained for VEGF-A (brown). Scale bar, 20 μm. (**B**) Percentages of VEGFR2^+^ area by counting positive cells in six randomly selected fields (400×) using Image Pro Plus 6.0 software. (**C**) mRNA levels of VEGF-A in tumor samples from WT and CD47^−/−^ mice (*n* = 4 per group). (**D**) Representative images of tumor sections stained for VEGFR2^+^ (brown). Scale bar, 20 μm. (**E**) Percentages of VEGFR2^+^ area by counting positive cells in six randomly selected fields (400×) using Image Pro Plus 6.0 software. (**F**) mRNA levels of VEGFR2 in tumor samples from WT and CD47^−/−^ mice (*n* = 4 per group). Data are mean ± SDs. *P < 0.05, **P < 0.01; ***P < 0.001.

VEGF is produced by many cell types including endothelial and tumor cells. VEGF produced by tumor cells can act in an autocrine fashion on tumor cells to promote tumorigenesis and to enhance tumor angiogenesis [24]. IHC analysis revealed that tumors from CD47-deficient mice had significantly increased expression of VEGF-A than those from WT mice (Figure 3A and 3B). Consistent with the protein levels, real time qPCR analysis showed a significantly increased expression of VEGF-A mRNA in the tumors from CD47-deficiet mice compared to those from WT mice (Figure 3C). Moreover, the levels of VEGFR2 in the tumors of CD47-deficient mice were significantly higher than in those from WT mice (Figure 3D and 3E). In addition, the levels of VEGFR2 mRNA levels in tumors grown in CD47-deficient mice were also significantly higher than those grown in WT mice (Figure 3F).

### Reduced TSP1 expression and macrophage recruitment in tumor from CD47-deficient mice

**Figure 4:**
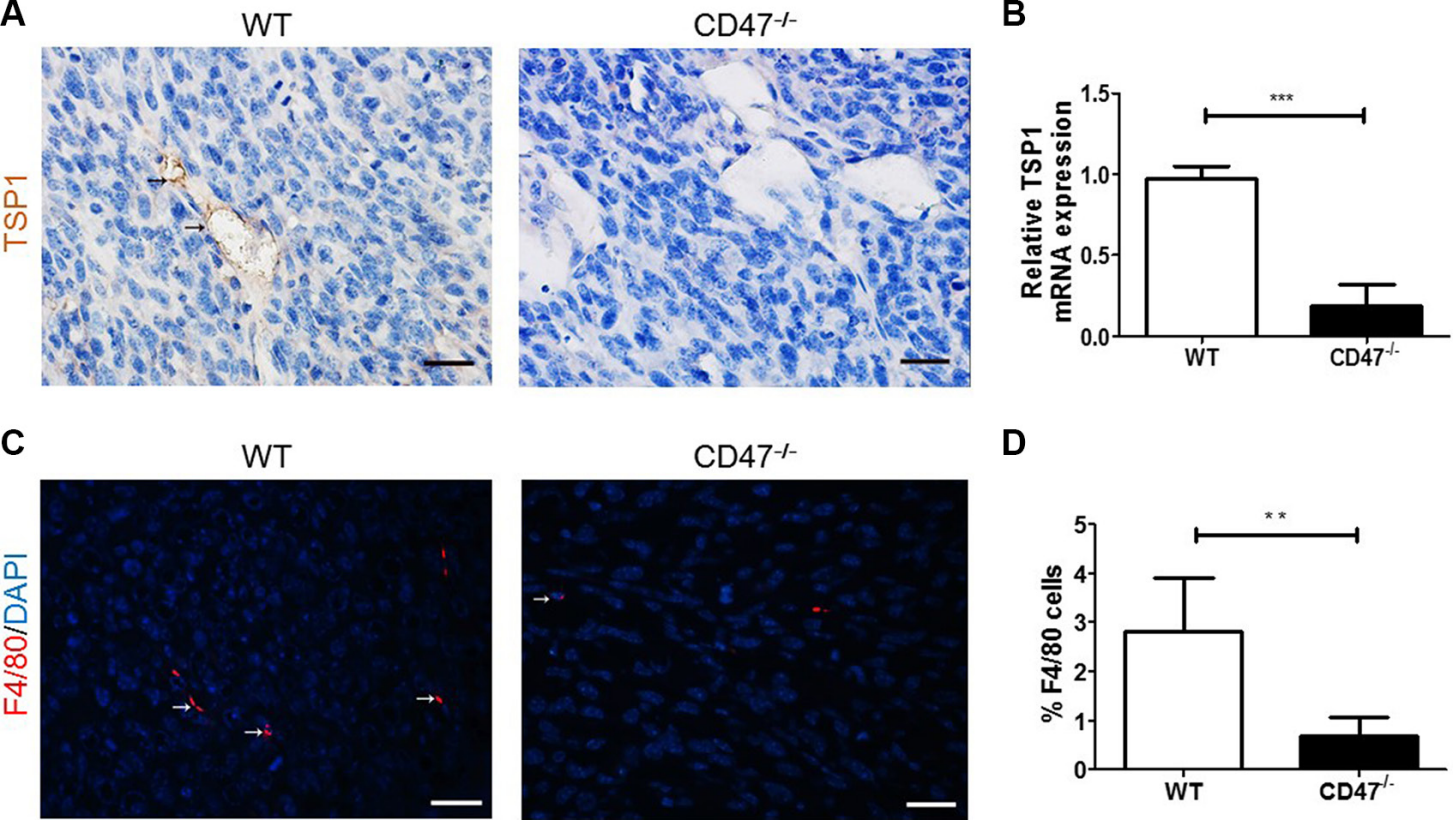
Reduced TSP1 production and the associated macrophage infiltration in tumors from CD47-deficient mice (**A**) Representative images of TSP1 staining (black arrow) of tumor removed from WT and CD47^−/−^ mice (Scale bar, 20 μm). (**B**) TSP1 mRNA levels in tumor samples from WT and CD47^−/−^ mice quantified by real-time PCR assay (*n* = 4/group). (**C**) Representative images of tumor sections stained for F4/80 (red; Scale bar, 20 μm). (**D**) Percentages of F4/80^+^ cells (*n* = 3/group). Data are mean ± SDs. **P < 0.01; ***P < 0.001.

TSP1 can be produced by both tumor cells [25] and endothelial cells [26]. To determine whether the improved angiogenesis in tumors from CD47-deficient mice was, at least in part, due to the lack of TSP1-CD47 signaling in endothelial cells, we measured TSP1 expression in the tumors. Tumors from CD47-deficient mice showed a significantly reduced TSP1 production, as measured by both IHC (Figure 4A) and real time qPCR (Figure 4B), compared to those from WT mice. Consistent with the role of TSP1 in recruiting macrophages into tumors [27], the number of F4/80^+^ tumor-infiltrating macrophages was significantly lower in CD47-deficient than WT mice (Figure 4C and 4D). These results suggested that CD47-deficency downregulated TSP1 production.

### Poor macrophage infiltration was attributed to reduced TSP1 production in the tumors in CD47-deficient mice

**Figure 5:**
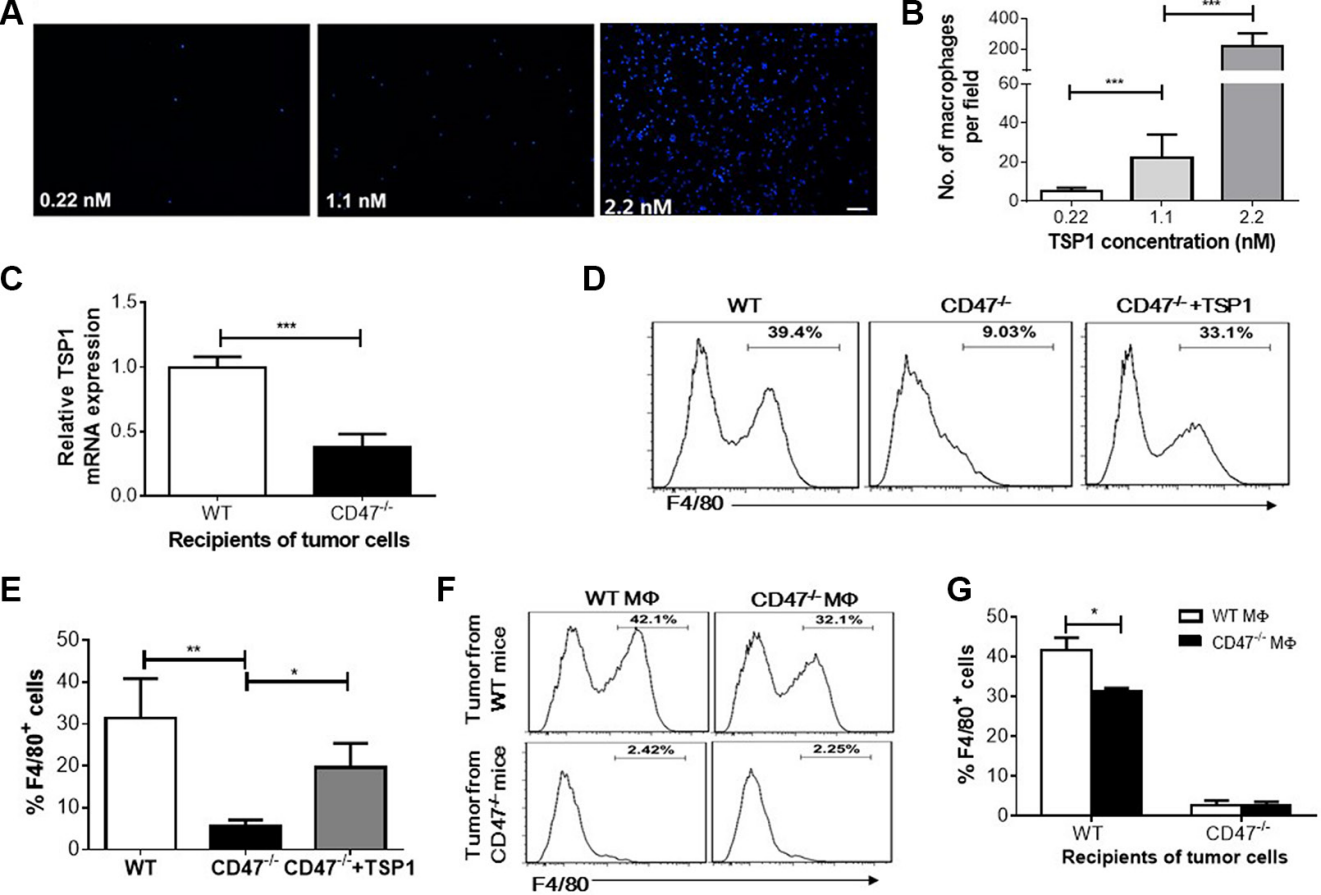
Macrophage recruitment by TSP1 and tumor cells *in vitro* (**A**–**B**) Dose-dependent recruitment of F4/80^+^ macrophages by TSP1 measured by Transwell assay. Shown are representative images of DAPI-stained macrophages (Scar bar, 50 μm) in the outer chamber (A) and numbers of macrophages per 100× microscopic filed (B). (**C**) TSP1 mRNA levels in 24-hr cultured tumor cells from WT and CD47^−/−^ mice (*n* = 4/group). (**D**–**E**) Recruitment of WT macrophages by cultured tumor cells from WT mice, CD47-deficient mice without (CD47^−/−^) or with (CD47^−/−^ + TSP1) recombinant mTSP1 (2.2 nM). Shown are representative flow cytometry profiles (D) and percentages (E) of F4/80^+^ macrophages in the outer chambers. (**F**–**G**) Recruitment of WT (WT MΦ) and CD47-deficient (CD47^−/−^ MΦ) macrophages by cultured tumor cells from WT or CD47-deficient mice. Shown are representative flow cytometry profiles (F) and percentages (G) of F4/80^+^ macrophages in the outer chamber of the transwell plate. Data are mean ± SDs. *P < 0.05, **P < 0.01; ***P < 0.001.

To test whether the reduced TSP1 production contributes to the inhibition of macrophage recruitment to tumor cells, we performed a macrophage migration assay using Transwell plates, in which TSP1 showed a dose-dependent effect on macrophage recruitment (Figure 5A and 5B). Tumor cell suspensions were prepared from WT or CD47-deficient mice, cultured for 20 hrs, and tested for the ability to recruit mouse macrophages using the Transwell plate system. We first confirmed that the level of TSP1 in the tumors from WT mice was significantly higher than that in tumors from CD47-deficient mice (Figure 5C), consistent with the data described above (Figure 4). The culture supernatant of the tumor cells from WT mice was significantly more effective in recruiting macrophages than that from CD47-deficient mice (Figure 5D and 5E). However, addition of TSP1 into the tumor cell culture supernatant from CD47-deficient mice significantly enhanced macrophage recruitment. Moreover, we found that although macrophages from CD47-deficient and WT mice exhibited relatively lower migration than WT macrophages towards tumor cell culture supernatants from WT mice, CD47-deficient macrophages showed significantly greater recruitment by the culture supernatant of tumor cells from WT mice (Figure 5F and 5G). These data indicate that TSP1 production in the tumor plays an important role in recruiting macrophages, and that the lower number of tumor-infiltrating macrophages in CD47-deficient mice is likely due to reduced TSP1 production in the tumor.

## DISCUSSION

Angiogenesis is a complex process depending on the coordination of many regulators by activating angiogenic switch [28]. Although TSP1 may potentially inhibit angiogenesis through its two receptors, CD47 and CD36, CD47-mediated signaling plays a more important role in response to physiological concentrations of TSP1 [29]. Binding of CD47 with SIRPα on macrophages and DCs conveys a “don't eat me” signal [11–14] and accordingly, the blockage of CD47-SIRPα binding induces tumor regression by activating macrophages [16] and DCs [18]. In this study, we demonstrated that CD47 expression on non-tumor cells, such as endothelial cells, plays a critical role in controlling tumor angiogenesis, and the lack of CD47 expression on endothelial cells significantly enhances angiogenesis, and effectively suppresses hypoxia-induced tumor necrosis and accelerates tumor progression.

VEGFs promote angiogenesis through multiple mechanisms, including facilitating endothelial cell invasion, migration and proliferation [30]. A previous study showed that TSP1 inhibits VEGFR2 signaling by disrupting the association of CD47 with VEGFR2 [10]. At the same time, TSP1 also antagonizes VEGF by inhibition of VEGF release from the extracellular matrix through suppression of MMP-9 activity [31]. We found that CD47 deficiency in endothelial cells enhances VEGF and VEGFR2 expression. This observation is consistent with the inhibitory role of TSP1-CD47 signaling in VEGFR expression. We also observed an increased VEGF production, which is in accordance with a decreased TSP1 production in CD47-deficient mice. Thus, the enhanced VEGF-VEGFR2 signaling may contribute to the observed enhancement of tumor angiogenesis in CD47-deficient mice. We also compared tumor growth and angiogenesis between WT and CD47^−/−^ tumor cells. Because CD47^−/−^ tumor cells are subject to immune rejection in WT mice, we injected these tumor cells into CD47-deficient mice and examined tumor growth and angiogenic potential within 9 days after tumor cell inoculation (Figure S1). We found no significant difference in tumor growth or angiogenesis between WT and CD47^−/−^tumors, supporting the potential of CD47 deficiency in tumor stroma to promote tumor progression by enhancing angiogenesis. In agreement with this, we recently showed that CD47 deficiency in both endothelial cells and the mouse microenvironment may promote angiogenesis [32].

TSP1 plays an important role in the recruitment of macrophages to injurious or inflammatory tissue [33, 34], and has been suggested to contribute to antitumor immunity by enhancing recruitment and activation of type-I (M1) macrophages [27]. We found that TSP1 production in tumors from CD47-deficient mice was significantly reduced compared to those in WT mice, likely due to markedly reduced HIF-1A, VEGF production and hypoxia-induced tumor necrosis. The associated inhibition of macrophage infiltration in the tumors from CD47-deficient mice supports the role of TSP1 in the recruitment of macrophages. To further support this, we found that the reduced macrophage infiltration in tumors from CD47-deficient mice is unlikely, primarily due to the defect of CD47-deficient macrophages, as both WT and CD47-deficient macrophages showed significantly increased migration to tumors from WT than to those from CD47-deficient mice.

Our data highlight a potentially overlooked effect of cancer immunotherapy using anti-CD47 antibodies. Although blocking the interaction between CD47 on tumor cells and SIRPα on the host macrophages and DCs induces antitumor immune responses, simultaneous interruption of the TSP1-CD47 signaling in tumor stromal cells, such as endothelial cells, may potentially facilitate tumor progression. It warrants further investigation as to whether improved tumor vascularization enhances antitumor therapy by promoting delivery of effector cells or chemotherapeutic agents to the tumor.

## MATERIALS AND METHODS

### Animals

WT and CD47-deficient (CD47^−/−^) C57BL/6J (B6) mice were purchased from Jackson Laboratory (Bar Harbor, ME, USA). Protocols involving animal experiments were approved by the Subcommittee on Research Animal Care of the First Hospital of Jilin University, and all of the experiments were performed in accordance with the protocols.

### Mouse tumor model

Tumor growth was induced by subcutaneous inoculation of a murine androgen insensitive prostate cancer RM1 cell line cells [35, 36], kindly provided by Dr. TC Thompson (MD Anderson Cancer Center). RM1 cells (1.0 × 10^6^ cells in 100 μl PBS) were inoculated in the groin of WT or CD47-deficient B6 mice. Tumor progression was determined by measuring tumor volume and tumor weight when mice were sacrificed at the indicated times. Tumor volume was calculated using the formula V = π × (d^2^ × D)/6 (d and D represents the minor and the major tumor axes, respectively). For histological analysis, tumor tissue was fixed in 4% paraformaldehyde at 4°C overnight, paraffin-embedded, and then sectioned in 4 μm thickness for hematoxylin and eosin (H&E) and immunohistochemistry (IHC) staining. Part of the tumor was frozen in liquid nitrogen and stored at −80°C for RNA and protein analyses.

### Immunohistochemistry (IHC)

Paraffin-embedded tissue sections (4 μm) were subjected to antigen retrieval, incubation with anti-CD31 (ab28364, abcam), anti-VEGF-A (BA0407, Boster), anti-HIF-1A (PB0245, Boster), anti-CD34 (BA0532, Boster), or anti-TSP1 (ab93653, abcam) antibodies, followed by incubation with a peroxidase-conjugated goat anti-rabbit IgG (KIT-9706, Maixin-Bio) secondary antibody. Samples were developed with DAB or AEC and counterstained with Mayor's hematoxylin (AR0005, Boster). Image Pro Plus 6.0 software was used for quantitative analysis.

### Immunofluorescence staining

Cryosections (4 μm) were incubated with antibodies against CD144 (550548, BD Pharmingen) or F4/80 (123115, BioLegend). Alexa Fluor 594-conjugated AffiniPure donkey anti-rat IgG (110988, Jackson ImmunoResearch) was used as the secondary antibody. Photomicrographs were produced using a fluorescent microscope (Olympus).

### Real time RT-PCR

Total RNA from transplanted tumor tissues was isolated with Multisource Total RNA Miniprep Kit (Axygen), and 1 μg was reverse-transcribed with random hexamer primer using the First-Strand cDNA Synthesis SuperMix (TransGen). According to the manufacturer's instructions, 5 ng cDNA was amplified in triplicate in a reaction volume of 10 μl with the StepOne Plus Real-time PCR System (Applied Biosystems) and StepOne Software v2.2.2 (Applied Biosystems). The expression level was normalized against the geometric mean of β-actin. Relative mRNA expression was calculated using the 2^−ΔΔCT^ method.

### Macrophage migration assay

The macrophage migration assay was performed using a multi-well chamber as the outer chamber and Transwell insert (8-μm pore-size) polycarbonate filter (BD Biosciences, Bedford, MA) as the inner chamber. Tumor cells prepared from WT or CD47-deficient mice were seeded in a 24-well plate (8 × 10^4^ per well), cultured for 20 h, then transferred to serum-free medium and plated into the outer chamber (8 × 10^4^/ml/well). Mouse peritoneal macrophages (labeled with F4/80-APC) were suspended in serum-free medium and seeded (8 × 10^5^ cells/200 μl/well) into the inner chamber. Where indicated, recombinant mouse TSP1 (R&D Systems) was added into the outer chamber. After incubation for 20 h at 37°C, the inner chamber was removed, and the cells in the outer chamber were harvested with trypsin and analyzed for the percentage of F4/80^+^ macrophages by flow cytometry. Cells were acquired using a BD LSRFortessa flow cytometer (BD Biosciences) and analyzed with FlowJo Software (7.2.1).

### Statistical analysis

All data are shown as mean ± SDs except for otherwise indicated. Significance was determined with two-tailed *t*-test analysis. The difference was considered significant when *P* ≤ 0.05.

## SUPPLEMENTARY MATERIALS FIGURES


