# Supplementary material for: CD47 deficiency in tumor stroma promotes tumor progression by enhancing angiogenesis
Source: Oncotarget. 2016 Jun 7;8(14):22406–13. doi: 10.18632/oncotarget.9899 (PMC5410232; doi:10.18632/oncotarget.9899)
Supplement: Supplementary file 1 [file oncotarget-08-22406-s001.pdf]

# CD47 deficiency in tumor stroma promotes tumor progression by enhancing angiogenesis

## Supplementary Materials

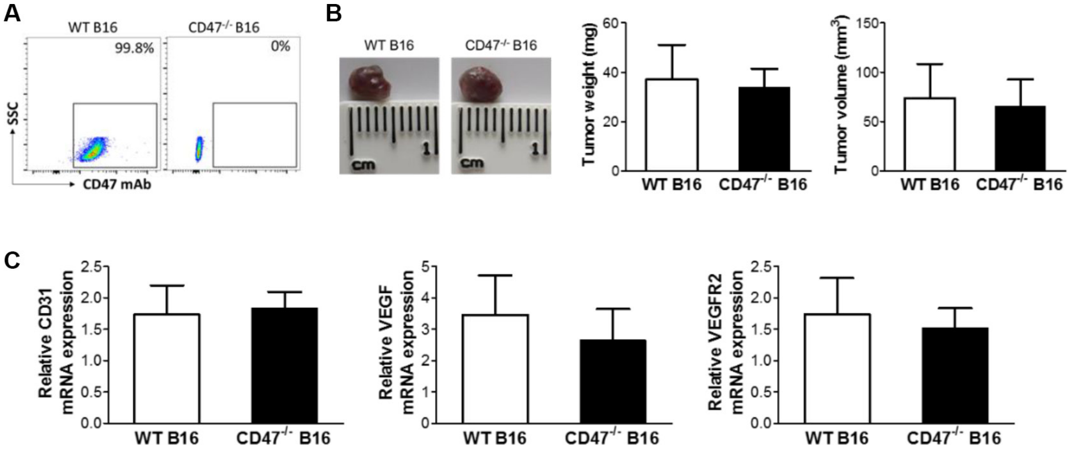


**Supplementary Figure S1: WT and CD47$^{-/-}$ melanoma cells show similar tumor growth and angiogenesis in CD47-deficient mice.** CD47$^{-/-}$ B16 melanoma cell line was generated by transient transfection of B16 cells with pSpCas9 (BB)-2A-GFP (PX458; a gift from Feng Zhang) [1], Addgene plasmid #48138 encoding a mouse CD47-targeting sgRNA (TTGGCGGCGGCGCTGTTGCT). (**A**) CD47 expression on WT and CD47$^{-/-}$ B16 cell lines. (**B**–**C**) WT and CD47$^{-/-}$ B16 cells were injected subcutaneously into CD47-deficient mice ($n$ = 9 per group), and tumors were harvested at day 9 post-tumor cell inoculation for measuring tumor growth and angiogenesis: (B) Representative tumor images (left), tumor weight (middle) and volume (right); (C) Relative expression levels of CD31, VEGF and VEGFR2 mRNAs in tumors quantified by real-time qPCR.

## REFERENCES

1. Ran FA, Hsu PD, Wright J, Agarwala V, Scott DA, Zhang F. Genome engineering using the CRISPR-Cas9 system. Nat Protoc. 2013; 8:2281–308.
